# The accuracy and precision of CT-RSA in arthroplasty: a systematic review and meta-analysis

**DOI:** 10.2340/17453674.2025.43334

**Published:** 2025-03-28

**Authors:** Sjors F VAN DE VUSSE, Nienke N DE LAAT, Lennard A KOSTER, Bart L KAPTEIN

**Affiliations:** Department of Orthopedics, Leiden University Medical Center, Leiden, The Netherlands

## Abstract

**Background and purpose:**

Computed tomography-based radiostereometric analysis (CT-RSA) is an alternative to conventional radiostereometric analysis (RSA) in measuring implant migration, circumventing the need for operative insertion of tantalum markers. The accuracy and precision of different CT-RSA techniques in various joints are still unclear, and the effective radiation dose (ED) of CT-RSA is usually higher than RSA. In this systematic literature review, we aimed to provide an overview of the accuracy, precision, clinical precision, and ED of CT-RSA techniques.

**Methods:**

We performed a systematic search in PubMed, Cochrane, and Embase databases. Main search items were “arthroplasty” AND “migration” AND “computed tomography.” We included full-text English papers, using CT for migration analysis (CT-RSA) in human, animal, or synthetic models with arthroplasties, reporting accuracy and/or precision. Eligible studies were screened and reviewed by 2 authors independently. Main outcomes were accuracy, precision, and clinical precision of CT-RSA in 6 degrees of freedom. Secondary outcome was the mean ED. A meta-analysis on (clinical) precision of CT-RSA was performed.

**Results:**

23 studies were included involving 163 patients, 20 human cadaveric, 3 porcine cadaveric, and 7 synthetic models. 6 different CT-RSA techniques were used to study 6 different joint components in cervical disc replacement and shoulder, hip, and knee arthroplasty. CT-RSA accuracy ranged between 0.02 and 0.71 mm and 0.03° and 1.00°. CT-RSA precision ranged between 0.00 and 0.47 mm and 0.00° and 1.09°. Mean precision was 0.15 mm (95% confidence interval [CI] 0.05–0.25) in the acetabulum, 0.13 mm (CI 0.00–0.28) and 0.24° (CI 0.00–0.51) in the proximal femur, and 0.04 mm (CI 0.00–0.08) and 0.07° (CI 0.00–0.15) in the proximal tibia. CT-RSA clinical precision ranged between 0.03 and 1.36 mm and 0.06° and 2.25°. Mean clinical precision was 0.13 mm (CI 0.11–0.16) and 0.26° (CI 0.20–0.32) in the acetabulum. The mean ED of CT-RSA ranged between 0.02 and 5.80 mSv.

**Conclusion:**

CT-RSA shows comparable accuracy and precision to standard RSA. CT-RSA seems to be a promising alternative to RSA.

Aseptic loosening after arthroplasties is the most frequent cause of implant failure and revision surgery [1-3]. It is caused by inadequate initial fixation of the implant after surgery, mechanical loss of fixation over time, or biological loss of fixation caused by particulate debris, leading to osteolysis [4]. In all cases, the loss of fixation results in micromotion between the implant and the bone. RSA is said to be a prognostic tool for predicting clinical aseptic loosening [5], as several studies have shown an association between early migration of an implant and later clinical aseptic loosening requiring revision surgery [6-9].

The current gold standard for detecting and analyzing migration of implants is radiostereometric analysis (RSA). The reported accuracy of measuring implant migration is between 0.05 and 0.50 mm for translations and between 0.15° and 1.15° for rotations [10-12]. Limitations of RSA include the requirement to insert tantalum markers in the bone during surgery, the need for a calibration system, and trained personnel for acquiring and processing the RSA radiographs [13,14].

In recent publications computed tomography-based radiostereometric analysis (CT-RSA) has been proposed as an alternative to RSA for implant migration measurement [15-18]. Feasibility studies using CT-RSA for analyzing implant migration of glenoid, femoral, and tibial components show comparable accuracy and precision to RSA [19,20]. However, in these studies, CT-RSA has a higher effective radiation dosage (ED) compared with RSA. For total hip arthroplasty, Brodén et al. estimated an ED for CT-RSA and RSA of 0.2 mSv and 0.04 mSv, respectively [21].

For RSA, accuracy, (in vitro) precision, and clinical precision have been established and extensively reported. However, a comprehensive overview of the accuracy, precision, clinical precision, and ED of CT-RSA is not available. We performed a systematic literature review to provide an overview of the accuracy, precision, clinical precision, and ED of CT-RSA for different CT-RSA techniques, CT protocols, and joints.

## Methods

We performed a systematic review following the Preferred Reporting Items for Systematic Reviews and Meta-Analyses (PRISMA) guidelines [22]. This review was registered in PROSPERO (ID: 444694) before databases were searched.

### Literature search

A search was performed in PubMed, Embase, and Cochrane databases. All literature available up until October 21, 2024 was searched. A medical librarian assisted with the search strategy. The main terms used in the search string were “arthroplasty” AND “migration,” AND “computed tomography,” along with their synonyms. We chose a broad search strategy to retrieve as many studies as possible. To prevent the exclusion of relevant studies we did not add study outcomes to the search string. The full search string is documented in the Supplementary data. Citation chaining did not result in additional studies.

### Inclusion and exclusion analysis

**Figure 1:**
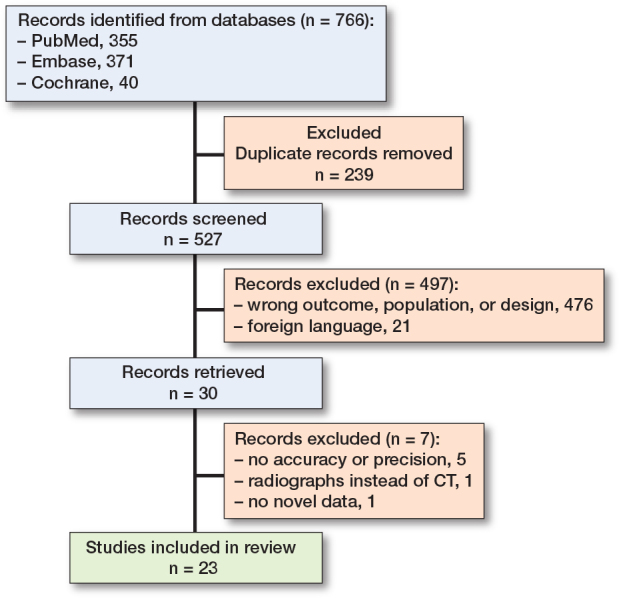
Flow diagram for the systematic review, which included searches of databases and registers [54].

Studies written in English and using CT for migration analysis (CT-RSA) in human, animal, or synthetic models with arthroplasties were included. Studies using imaging modalities other than CT, conference abstracts, and studies without novel data collection were excluded. Studies were included only if they reported either accuracy, measured using a test setup, or in-vitro/in-vivo precision, using double measurements under unchanged conditions. 2 authors (SV and NL) independently screened the title and abstract for selection criteria using Rayyan software [23] (Figure 1). Inclusion of studies was decided after full-text screening. If no consensus decision could be made, all authors decided on possible inclusion.

### Data extraction

The primary outcomes extracted were accuracy, precision, and clinical precision of implant migration in millimeter translation (mm) and degree rotation (°). When available, total translation (TT) was also collected. The secondary outcome was the mean ED in mSv. Data extraction further included study design, joint type, CT scanner, pixel size, slice thickness, CT-RSA technique, implant type, origin of the bone model, and migration coordinate system. Accuracy is defined by trueness and precision [24]. Trueness is the measure of agreement between the mean value (obtained from a large series of test results) and an accepted reference value [25]. Precision is the closeness of agreement between independent test results obtained under unchanged conditions [25]. When precision is determined in a clinical setting using double examinations, this is called clinical precision. The guideline by Kaptein et al. recommends describing accuracy by both bias (trueness) and standard deviation (precision) [25]. However, in most papers accuracy was given in root mean square error (RMS), which is the square root of the average squared errors. We chose to describe accuracy results accordingly, as RMS combines bias and standard deviation. Note that in absence of bias, RMS is the same as SD for normally distributed data. Additionally, we chose to describe the precision, in-vitro and in-vivo, as the standard deviation (SD). To calculate the upper limit of the 95% confidence interval of the accuracy and precision, RMS or SD respectively should be multiplied by 1.96. However, when the sample size (N) is small (< 30), multiplying by the corresponding t-value according to Student’s t-distribution table is considered to give a better estimation [26,27]. Migration of an implant with respect to the bone in CT measurements is, for a right-sided implant, expressed as medial (Tx) translation, posterior (Ty) translation, proximal (Tz) translation, and rotations (Rx, Ry, Rz) about these axes. The default CT-RSA coordinate system differs from RSA [25]. Therefore, we adjusted all results to be compatible with the CT coordinate system described by Kaptein et al. [25].

### Data synthesis and analysis

Study characteristics were used to compile an overview of the different CT protocols and study setups. Accuracy and (clinical) precision of all CT-RSA techniques were systematically reported in outcome-specific tables to compare differences in CT-RSA techniques, joints, and axes. The accuracy and precision of micro-CT techniques were reported separately.

If possible, raw data was used to recalculate outcomes for accuracy and precision when presented differently in the original study. In 4 studies we recalculated the accuracy and precision ourselves, once with received data from the authors [28] and 3 times with reported data from the paper [17,29,30]. Raw data was unavailable for 3 studies. In those studies, we reported the original outcomes [17,31,32]. In 3 studies the specification of migration measurement and axes definition was missing [29,33,34]. Unfortunately, no additional information was obtained from contacting the authors. We reported the ED for both in-vitro and in-vivo studies for all joints.

We performed meta-analyses for different joints and implant components separately, combining all CT-RSA techniques (except for micro-CT) in the analysis. In agreement with Pijls et al. (2012), we pooled data if results of 3 or more studies were available [7]. We calculated the respective mean outcome and standard error (SE) of all eligible studies, combining x, y, and z measurements, for translations and rotations separately. We reported results in means and 95% confidence intervals (CI). In addition, we calculated 95% prediction intervals (PI) for subgroups [35]. Meta-analysis was performed using RevMan 5.4 software (https://test-training.cochrane.org/online-learning/core-software-cochrane-reviews/review-manager-revman/download-revman-5), which provides an I^2^ index, indicating heterogeneity of the pooled studies.

### Risk of bias

All included articles were appraised using the Critical Appraisal Skills Programme (CASP) for diagnostic test studies [36]. Quality assessment was undertaken by the first author (SV) and confirmed by all authors. Quality of studies ranged between 55% and 100%, with a median of 91% (Table 7, see Supplementary data).

### Funding, use of AI tools, and disclosures

Authors did not receive funding for the paper. Furthermore, no AI tools were used in writing this paper.

Complete disclosure of interest forms according to ICMJE are available on the article page, doi: 10.2340/17453674.2025.43334

## Results

527 studies were identified, of which 23 met the inclusion criteria [15-18,21,28-34,37-47]. For the assessment of CT-RSA in a clinical setting, 8 cohorts included 163 patients undergoing hip arthroplasty and cervical disc replacement. For the assessment of accuracy and precision in an in-vitro setting, 17 studies included 20 human cadaveric, 3 porcine cadaveric, and 7 synthetic models in cervical disc replacement and shoulder, hip, and knee arthroplasty. 6 different CT-RSA techniques were used, examining cervical disc, glenoid, humeral, acetabular, femoral hip, and tibial knee components. 4 studies used a micro-CT scanner, which differed from conventional CT scanners in terms of scan protocol and voxel size, and thus the resolution. Studies using a conventional CT scanner had voxel sizes ranging between 0.16 and 1.00 mm, compared with 0.02 and 0.04 mm for micro-CT scanners (Table 1).

**Table 1:** Study characteristics

Author	CT-RSA	CT scanner	Pixel size (mm)	Slice thickness (mm)	Model (sample)	Joint	Implant (cementation)	Markers (n)	Statistics ^a^	ED (mSv)
Olivecrona (2003)	3D volume tool	Lightspeed	–	1.25	Synthetic (1)	Hip (acetabulum)	Zimmer Biomet (unknown)	–	A) t*RMS P) t*SD C) –	–
Gortchacow (2011)	micro-CT- technique	μCT	–	–	Human cadaveric (1)	Hip (femur)	Symbios (cementless)	+ (8)	A) – P) t*SD C) –	–
Svedmark (2011) **^b^**	3D volume	1) Lightspeed 2) Somatom	1) 0.39 2) 1.00	1) 0.50 2) 0.22	1) Synthetic (1) 2) Human (9)	Spine (cervical disc)	DePuy Synthes (unknown)	–	A) t*RMS P) t*SD C) t*SD	0.33
Gortchacow (2012)	micro-CT- technique	μCT	0.04 (voxel)	0.04 (voxel)	Human cadaveric (6)	Hip (femur)	Symbios (cementless)	+ (12)	A) – P) t*SD C) –	–

Sukjamsri (2015)	micro-CT- technique	μCT	0.02 (voxel)	0.02 (voxel)	Porcine cadaveric (1)	Shoulder (glenoid)	Mathys Ltd (cementless)	–	A) t*RMS P) t*SD C) –	–

Boettner (2015)	Geomagic Studio 7	Discovery	-	0.80	Human cadaveric (2)	Hip (femur)	Stryker (cementless)	+ (6)	A) t*RMS P) t*SD C) –	3.80 1.60 0.70
Boettner (2016)	Geomagic Studio 7	–	–	0.80	Human cadaveric (3)	Hip (femur)	Unknown (cementless)	+ (6)	A) t*RMS P) t*SD C) –	3.20
Malfroy Camine (2016)	micro-CT- technique	μCT	0.04 (voxel)	0.04 (voxel)	Human cadaveric (1)	Hip (femur)	DePuy Synthes (cementless)	+ (30)	A) – P) t*SD C) –	–
Scheerlinck (2016)	CTSA	Somatom	0.18	0.60	Human cadaveric (1) Human (5)	Hip (femur)	Zimmer Biomet (cemented)	–	A) MAE P) t*SD C) MAE	5.80 5.50
Brodén (2016)	3D volume tool	Discovery	0.60	0.60	Synthetic (2)	Hip (1) (acetabulum)	1) Waldemar Link (cementless) 2) Biomet (cemented)	+ (39)	A) – P) t*SD C) –	0.33
Eriksson (2019)	CTMA	Somatom	0.13	0.60	Synthetic (1)	Hip (femur)	Zimmer Biomet (cementless)	+ (9)	A) – P) t*SD C) –	0.70
Brodén, Sandberg (2020) **^c^**	CTMA	(1) Somatom (2) Discovery (3) Aquilion One	–	1) 0.60 2) 0.63 3) 0.50	Human (24)	Hip (acetabulum)	(1,3) Waldemar Link (cemented) (2) Biomet (cementless)	–	A) – P) – C) t*SD	0.70 0.20 2.30
Brodén, Giles (2020)	CTMA	Ingenuity	0.65	1.00	Human cadaveric (1)	Shoulder (glenoid; humerus)	Mathys Ltd (cementless)	+ (21)	A) t*RMS P) t*SD C) –	0.27
Sandberg (2020)	CTMA	Somatom	0.13	0.60	Human (20)	Hip (acetabulum; femur)	Waldemar Link (unknown)	+ (–)	A) – P) – C) t*SD	1.70
Stigbrand (2020)	CTMA	Aquilion One	–	–	Human (17)	Hip (acetabulum)	Waldemar Link (cemented)	–	A) – P) – C) t*SD	2.30
Brodén (2021)	CTMA	Discovery	–	0.63	Human (10)	Hip (acetabulum)	Zimmer Biomet (unknown)	–	A) – P) – C) t*SD	0.20
Jun (2022)	3D volume tool	Somatom	-	0.60	Synthetic (1)	Shoulder (glenoid)	DePuy Synthes (unknown)	+ (3)	A) t*RMS P) – C) –	–
Angelomenos (2022)	CTMA	Somatom	-	0.63	Human (30)	Hip (acetabulum)	Zimmer Biomet (cemented)	–	A) – P) – C) t*SD	0.80
Clarke (2023)	CTSA	Somatom	0.34	0.60	Synthetic (1)	Hip (acetabulum; femur)	Embody Ltd (cementless)	+ (18)	A) t*RMS P) t*SD C) –	0.25
Engseth (2023) **^d^**	CTMA	(1) GE Revolution (2) Somatom	0.39	0.63	Porcine cadaveric (1)	Knee (tibia)	Zimmer Biomet (unknown)	+ (14)	A) – P) t*SD C) –	0.08
Øhrn (2023)	CTMA	GE Revolution	–	0.63	Porcine cadaveric (1)	Knee (tibia)	Zimmer Biomet (unknown)	–	A) – P) t*SD C) –	0.02
Polus (2024)	V3MA	Aquilion One	0.45	0.50	Human (48)	Hip (femur)	DePuy Synthes (cementless)	–	A) – P) – C) t*SD	1.51
De Laat (2024)	V3MA	Aquilion One	0.30	0.50	Human cadaveric (5)	Knee (tibia)	Zimmer Biomet (both)	–	A) t*RMS P) t*SD C) –	0.16

Footnote Table 1: CT-RSA = computed-tomography based roentgen stereophotogrammetric analysis; mm = millimeter; CTSA = computed-tomography spatial analysis; CTMA = computed tomography-based micromotion analysis; V3MA = volumetric matching micromotion analysis; t = t-value; SD = standard deviation, RMS = root mean square, MAE = mean absolute error.

a Statistical formulas are presented in the order of accuracy (A), precision (P), and clinical precision (C). If the outcome was not available, ‘–’ was noted.

b Protocol (1) uses a model for the in-vitro measurement of total cervical disc migration. Protocol (2) uses 9 patients for in-vivo measurements.

c The study uses clinical data from 3 different hospitals in Sweden: (1) Uppsala University Hospital, (2) Danderyds Hospital, and (3) Gävle Hospital.

d Protocols (1) and (2) use different CT scanners.

### Reported accuracy of CT-RSA

10 studies performed accuracy measurements in an in-vitro test setup (Table 2). Of these, 9 CT-RSA accuracy studies used a conventional CT scanner and 1 a micro-CT scanner. In these studies, the predefined migration of a phantom model was simulated and compared with measured migration using a CT-RSA technique. Combining all CT-RSA techniques using conventional CT, the accuracy ranged between 0.02 and 0.71 mm and 0.03° and 1.00°. In 2 studies the accuracy of total translation for femoral head components was available, which ranged between 0.11 and 0.23 mm. The micro-CT study showed accuracy ranging between 0.03 and 0.12 μm for glenoid components.

**Table 2:** Reported accuracy of CT-RSA

Joint/Method	Author	n	Tx	Ty	Tz	Rx	Ry	Rz	U	TT
Acetabulum										
CTSA	Clarke (2023)	T17;R15	0.08	0.06	0.04	0.17	0.29	0.43	–	–
3D volume tool	Olivecrona (2003)	T30	–	–	–	–	–	–	T0.61	–
Proximal femur										
CTSA	Clarke (2023)	T17;R15	0.18	0.04	0.15	0.28	0.46	0.36	–	–
	Scheerlinck (2016)	T39;R39	0.05	0.04	0.03	0.04	0.08	0.06	–	–
Geomagic 7	Boettner (2015) **^a^**	1) T30	–	–	–	–	–	–	–	0.23
			2) T30	–	–	–	–	–	–	–
			3) T30	–	–	–	–	–	–	–
	Boettner (2016)	T15	–	–	–	–	–	–	–	0.11
Tibia										
V3MA	De Laat (2024)	T9;R6	0.05	0.02	0.15	–	–	0.03	–	–
Glenoid										
CTMA	Brodén, Giles (2020)	T16;R12	0.23	0.17	0.20	0.44	0.48	0.71	–	–
micro-CT	Sukjamsri (2015)	T12	0.12	0.03	0.07	–	–	–	–	–
3D volume tool	Jun (2022)	T9;R3	0.06	0.24	0.15	–	–	0.11	–	–
Humerus										
CTMA	Brodén, Giles (2020)	T16;R12	0.11	0.07	0.09	0.34	0.32	0.22	–	–
Spine										
3D volume tool	Svedmark (2011)	T61;R61	0.57	0.28	0.71	1.00	0.57	0.28	–	–

For abbreviations, see Table 1.

n is the number of measurements with ‘T’ as translations and ‘R’ as rotations. Studies that used multiple protocols were named (1), (2), etc. Axis definition is reported according to the CT coordinate system: medial (X), posterior (Y), and proximal (Z). Measurements with unknown axis definition are depicted in U. Measurements in all axes are shown in mm for translations and degrees for rotations, and respectively in μm and millidegrees for micro-CT. If available, total translation (in mm) was inserted in TT.

a 3 different radiation protocols were used: (1) standard protocol for hip implants, (2) and (3) alternative low-dose protocols.

### Reported precision of CT-RSA

16 studies performed in-vitro precision measurements in a test setup using repeated examinations (Table 3). The phantom models were repositioned between scans, while the conditions remained unchanged. 12 conventional CT studies showed a combined precision ranging between 0.00 and 0.47 mm and 0.00° and 1.09°. Meta-analysis showed a mean precision of 0.15 mm (CI 0.05–0.25 and PI –0.16 to 0.46) for the acetabulum, 0.13 mm (CI 0.00–0.28 and PI –0.21 to 0.47) and 0.24° (CI 0.00–0.51 and PI –0.31 to 0.79) for the proximal femur, and 0.04 mm (CI 0.00–0.08 and PI –0.01 to 0.09) and 0.07° (CI 0.00–0.15 and PI -0.01 to 0.15) for the proximal tibia (Table 4). Heterogeneity between pooled studies was high, with an I^2^ index between 93 and 99% (Figure 2, see Supplementary data).

**Table 3:** Reported precision of CT-RSA

Joint/Method	Author	n	Tx	Ty	Tz	Rx	Ry	Rz	U	TT
Acetabulum										
CTSA	Clarke (2023)	9	0.08	0.09	0.11	0.38	0.42	0.19	–	–
3D volume tool	Olivecrona (2003)	7	–	–	–	–	–	–	T0.47	–
	Brodén (2016) **^a^**	1) 6	0.01	0.09	0.04	0.10	0.21	0.06	–	–
		2) 6	0.04	0.06	0.04	0.14	0.27	0.29	–	–
Proximal femur										
CTMA	Eriksson (2019)	4	0.14	0.25	0.14	0.47	0.11	0.42	–	–
CTSA	Scheerlinck (2016)	8	0.01	0.00	0.03	0.00	0.00	0.05	–	–
	Clarke (2023)	9	0.27	0.11	0.28	0.42	0.34	0.41	–	–
micro-CT	Gortchacow (2011)	18	5.00	5.00	9.00	4.70	4.70	3.70	–	–
	Gortchacow (2012)	6	–	–	–	–	–	–	T15,00	–
	M. Camine (2016)	3	4.70	5.10	3.70	–	–	–	–	–
Geomagic 7	Boettner (2015) **^b^**	1) 30	–	–	–	–	–	–	–	0.22
		2) 30	–	–	–	–	–	–	–	0.18
		3) 30	–	–	–	–	–	–	–	0.20
	Boettner (2016)	15	–	–	–	–	–	–	–	0.13
Tibia										
CTMA	Engseth (2023) **^c^**	1) 21	0.06	0.03	0.08	0.04	0.09	0.07	–	0.04
		2) 21	0.05	0.09	0.09	0.21	0.06	0.13	–	0.08
	Øhrn (2023)	21	0.01	0.01	0.01	0.01	0.01	0.01	–	0.01
V3MA	De Laat (2024)	16	0.01	0.02	0.06	0.02	0.02	0.07	–	–
Glenoid										
CTMA	Brodén, Giles (2020)	28	0.15	0.13	0.13	0.33	0.38	0.54	–	–
micro-CT	Sukjamsri (2015)	5	–	–	13.00	–	–	–	–	–
Humerus										
CTMA	Brodén, Giles (2020)	28	0.08	0.09	0.11	0.26	0.27	0.23	–	–
Spine										
3D volume tool	Svedmark (2011)	13	0.18	0.29	0.22	1.09	0.63	0.92	–	–

For abbreviations: see Tables 1 and 2

a 2 models were used: 1) uncemented implant and 2) cemented implant.

b 3 different radiation protocols were used: 1) standard CT protocol for hip implants, 2) and 3) low-dose CT protocols.

c Protocol 1) uses a GE Revolution CT scanner and 2) a Somatom CT scanner.

**Table 4:** Pooled estimates of subgroups. Translations are in millimeters and rotations in degrees

Joint component Outcome	No. of studies	Mean precision	CI	PI
Acetabulum				
In-vitro, translations	3	0.15	0.05 to 0.25	–0.16 to 0.46
In-vivo, translations	7	0.13	0.11 to 0.16	0.10 to 0.16
In-vivo, rotations	6	0.26	0.20 to 0.32	0.20 to 0.32
Proximal femur				
In-vitro, translations	3	0.13	0.00 to 0.28	–0.21 to 0.47
In-vitro, rotations	3	0.24	0.00 to 0.51	–0.31 to 0.79
Proximal tibia				
In-vitro, translations	3	0.04	0.00 to 0.08	–0.01 to 0.09
In-vitro, rotations	3	0.07	0.00 to 0.15	–0.01 to 0.15

CI = 95% confidence interval, PI = 95% prediction interval

In 4 studies the precision of total translation was available. For femoral head components TT ranged between 0.13 and 0.22 mm and for tibial knee components between 0.01 and 0.08 mm. The 4 micro-CT studies showed a precision ranging between 3.7 and 15.0 μm and 3.7 and 4.7 millidegrees for femoral hip components, and 13.0 μm for glenoid components.

### Reported clinical precision of CT-RSA

8 studies performed in-vivo precision measurements using double examinations (Table 5). Patients were repositioned between scans, while their conditions remained unchanged. These clinical CT studies showed in-vivo precision ranging between 0.03 and 1.36 mm and 0.06° and 2.25°. Meta-analysis showed a mean clinical precision of 0.13 mm (CI 0.11–0.16 and PI 0.10–0.16) and 0.26° (CI 0.20–0.32 and PI 0.20–0.32) in the acetabulum (Table 4). The I^2^ index ranged between 68 and 80% (Figure 2, see Supplementary data).

**Table 5:** The reported clinical precision of CT-RSA

Joint/Method	Author	n	Tx	Ty	Tz	Rx	Ry	Rz	U
Acetabulum									
CTMA	Sandberg (2020)	9	0.07	0.17	0.14	0.19	0.21	0.07	–
	Stigbrand (2020)	12	–	–	–	–	–	–	T0.11–0.14
	Brodén, Sandberg (2020) **^a^**	1) 5	0.07	0.13	0.31	0.37	0.22	0.39	–
		2) 9	0.23	0.11	0.08	0.31	0.28	0.29	–
		3) 10	0.12	0.31	0.15	0.28	0.20	0.23	–
	Brodén (2021)	10	0.16	0.14	0.10	0.25	0.21	0.31	–
	Angelomenos (2022)	20	0.06	0.08	0.13	0.33	0.23	0.35	–
Proximal femur									
CTMA	Sandberg (2020) **^b^**	1) 9	0.09	0.24	0.06	0.08	0.06	0.36	–
		2) 9	0.06	0.07	0.07	0.08	0.06	0.36	–
		3) 9	0.19	0.17	0.07	0.08	0.06	0.36	–
CTSA	Scheerlinck (2016)	5	–	–	–	–	–	–	T0.19;R0.23
V3MA	Polus (2024)	48	0.05	0.03	0.13	0.06	0.06	0.22	–
Spine									
3D volume tool	Svedmark (2011)	7	1.36	0.40	0.29	2.25	0.47	1.78	–

For abbreviations: see Table 1 and 2.

a The study uses clinical data from 3 different hospitals in Sweden: 1) Uppsala University Hospital, 2) Danderyds Hospital and 3) Gävle Hospital.

b The study provides precision measurements of the 1) head, 2) neck, and 3) tip of the femoral implant

### Reported ED of CT-RSA

The ED of the in-vitro studies ranged between 0.02 and 5.80 mSv. The in-vivo ED of all joints ranged between 0.20 and 5.50 mSv (Table 6). The ED was highest in the hip joint, with a mean ED ranging between 0.20 and 2.30 mSv for the acetabulum and 1.32 and 5.50 mSv for the proximal femur in clinical studies.

**Table 6:** The reported effective dosage (ED) in mSv of CT-RSA for each examined joint for in-vitro and in-vivo studies

	No. of studies	ED (min-max)
In-vitro		
Cervical disc	1	0.33
Glenoid	1	0.27
Proximal humerus	1	0.27
Acetabulum	2	0.25–0.33
Proximal femur	7	0.25–5.80
Proximal tibia	3	0.02–0.16
All joints	15	0.02–5.80
In-vivo		
Cervical disc	1	0.33
Acetabulum	7	0.20–2.30
Proximal femur	4	1.32–5.50
All joints	12	0.20–5.50

## Discussion

This systematic review was performed to provide an overview of the accuracy and (clinical) precision using different CT-RSA techniques on implant migration. We showed that CT-RSA had comparable accuracy and precision to standard RSA. Micro-CT techniques were more accurate and precise than conventional CT techniques.

In CT-RSA the hip joint had the highest effective dose (ED), due to its higher tissue weight factor [48]. Sandberg et al. suggest that CT protocols with an ED below 1 mSv may be considered as low-dose protocols [27]. According to EU guidelines CT-RSA usually falls in Category IIa, where a maximum ED of 10 mSv in patients older than 50 years is accepted for the entire duration of the study [50]. In a typical 2-year hip migration study with 5 examinations, CT-RSA has a total ED of 5*0.2 = 1.0 mSv using the lowest clinical dose protocol for the acetabulum, which is still below the acceptable threshold, but higher than the ED of 5*0.04 = 0.2 mSv for a similar RSA study of the hip joint [21,49]. Therefore, low-dose CT protocols should be used to reduce effective dose, because low-dose protocols showed comparable accuracy and (clinical) precision to standard protocols [15,16,21,32].

CT scan protocols varied, resulting in voxel sizes between 0.16 and 1.00 mm. Accuracy and precision results in micro-CT were better, with voxel sizes ranging between 0.02 and 0.04 mm, which suggests that a smaller pixel size and slice thickness leads to more accurate and precise results. However, the use of micro-CT techniques for in-vivo implant migration measurements is not yet practically feasible, due to technical limitations and higher radiation exposure [51]. Nevertheless, micro-CT can already play a role in validation studies and implant design testing. The question remains whether micro-CT is necessary, considering that accuracy and precision from conventional CT is good enough for clinical migration measurements.

Two different techniques are used in CT-RSA: surface registration, such as in CT-based Micromotion Analysis (CTMA; Sectra Medical, Linköping, Sweden) software and image volume registration, such as in CT-based Spatial Analysis (CTSA) techniques [17,43], and Volumetric Matching Micro Motion Analysis (V3MA; RSAcore, Leiden, The Netherlands) software [44,46]. The reported data did not show differences in outcomes for these techniques. Surface registration can either be marker-based [42,52] or marker-free [15,19]. Some studies used tantalum bone-markers to reconstruct the rigid body, which did not result in measurably different accuracy or precision, showing that, unlike in RSA, markers are not necessary in CT-RSA. The developments in artificial intelligence are promising to further automate some of the manual work that is still necessary in CT-RSA analysis [53].

### Limitations

Regarding the literature search string, we omitted the outcomes as a search parameter in the used search string to acquire more studies and therefore prevent the exclusion of relevant studies. We did not include accuracy measurements in a clinical setting, as the “true” migration is unknown. With in-vitro accuracy studies, a pre-defined (true) migration is known, which allows estimation of the accuracy of a CT-RSA technique in measuring migration. Some studies used RSA measurements as true migration in clinical studies; however, although RSA is currently the gold standard, it is not a reliable reference value as it also has measurement errors. Therefore, we did not include accuracy results of these papers [42,53]. Furthermore, we investigated migration in 6 degrees of freedom, as accuracy and precision can differ for different axes. Generally, accuracy, precision, and clinical precision were found to be best around the posterior–anterior axis, although differences are small.

Unfortunately, 10 studies did not provide complete migration data. In 4 we recalculated the outcomes using provided [17,29,30] or received [28] raw migration data. We contacted authors for missing migration data, but most did not respond or provide information. Consequently, some studies used different formulas or lacked axis definitions. In addition, in-vivo studies examined only hip and cervical disc component migration, so clinical precision results in other joints remain uncertain.

We performed a meta-analysis if results of 3 or more studies per joint component were available. This meant that we could not pool accuracy results. We did pool in-vitro and in-vivo precision results when possible. Heterogeneity between studies was high, probably due to small sample sizes and range of results. Due to high heterogeneity and the limited number of studies, these findings should be considered critically. To better interpret these results clinically, we calculated the 95% prediction interval. The 95% predication interval estimates where the true effects of an intervention are likely to fall in 95% of future comparable studies. If there is no heterogeneity between studies, the prediction interval will be the same as the confidence interval. However, when heterogeneity is present, the PI will be wider than the CI [35].

### Conclusion

Based on the currently available literature, assessing different arthroplasties and cervical disc replacement using different CT scanners, protocols, and migration analysis software, CT-RSA is generally comparable to RSA in accuracy, precision, and clinical precision. Furthermore, CT-RSA has practical advantages, suggesting CT-RSA is a feasible alternative to RSA.

*In perspective,* future research should focus on further implementation in clinical follow-up studies with low-dose CT protocols. Also, future studies should focus on (long-term) clinical precision in joints other than the hip and the spine. In arthroplasty of the hand and foot the volume in which RSA markers can be placed is limited. CT-RSA could be a suitable solution for measuring migration in these areas, provided that the accuracy and precision have been established in multiple studies. Radiation for CT-RSA is higher than RSA, but low-dose protocols are still within the classification of acceptable in studies according to EU guidelines.

### Supplementary data

Figure 2, Table 7, and search strings are available as Supplementary data on the article page, doi: 10.2340/17453674.2025.43334

## Supplementary Material



## Notes

NL initiated the project. BK coordinated the project. SV and NL carried out study selection and data extraction and wrote the initial draft. All authors revised the manuscript.

The authors would like to thank Claudia Pees, medical librarian, for her help in creating the search string for all databases. Furthermore, the authors would like to thank Bart Pijls for his advice regarding the meta-analysis.

Handling co-editors: Li Felländer-Tsai and Robin Christensen

*Acta* thanks Hannu T Aro and Petri Virolainen for help with peer review of this manuscript.
