# The accuracy and precision of CT-RSA in arthroplasty: a systematic review and meta-analysis

**DOI:** 10.2340/17453674.2025.43900

**Published:** 2025-05-27

**Authors:** Sjors F VAN DE VUSSE, Nienke N DE LAAT, Lennard A KOSTER, Bart L KAPTEIN

**Affiliations:** Department of Orthopedics, Leiden University Medical Center, Leiden, The Netherlands

To our unpleasant surprise, we discovered that we have mistakenly included incorrect values in Table 2 (Reported accuracy of CT-RSA) for the paper by De Laat et al. (2024).

The paper by De Laat (2024) is from our group as well. In the draft version of the systematic review manuscript, we included data from a draft manuscript of the paper by De Laat. Before publication of De Laat (2024) we updated the values for the RMS error for accuracy in translations and rotations based on an increased number of observations. However, we have overlooked to use the updated values for Table 2 of the systematic review. We are sincerely sorry for this mistake.

We believe it is important to be transparent and to ensure a correct link between the systematic review and the paper by De Laat. These requested changes do not alter our conclusions of the systematic review.

On behalf of all authors

Bart Kaptein

## Errorneous version

### Reported accuracy of CT-RSA

10 studies performed accuracy measurements in an in-vitro test setup (Table 2). Of these, 9 CT-RSA accuracy studies used a conventional CT scanner and 1 a micro-CT scanner. In these studies, the predefined migration of a phantom model was simulated and compared with measured migration using a CT-RSA technique. Combining all CT-RSA techniques using conventional CT, the accuracy ranged between 0.02 and 0.71 mm and 0.03° and 1.00°. In 2 studies the accuracy of total translation for femoral head components was available, which ranged between 0.11 and 0.23 mm. The micro-CT study showed accuracy ranging between 0.03 and 0.12 µm for glenoid components.

## Corrected version

### Reported accuracy of CT-RSA

10 studies performed accuracy measurements in an in-vitro test setup (Table 2). Of these, 9 CT-RSA accuracy studies used a conventional CT scanner and 1 a micro-CT scanner. In these studies, the predefined migration of a phantom model was simulated and compared with measured migration using a CT-RSA technique. Combining all CT-RSA techniques using conventional CT, the accuracy ranged between **0.03** and 0.71 mm and **0.04°** and 1.00°. In 2 studies the accuracy of total translation for femoral head components was available, which ranged between 0.11 and 0.23 mm. The micro-CT study showed accuracy ranging between 0.03 and 0.12 µm for glenoid components.

## Errorneous version

**Table 2:** Reported accuracy of CT-RSA

Joint/Method	Author	N	Tx	Ty	Tz	Rx	Ry	Rz	U	TT
Acetabulum										
CTSA	Clarke (2023)	T17;R15	0.08	0.06	0.04	0.17	0.29	0.43	–	–
3D volume tool	Olivecrona (2003)	T30	–	–	–	–	–	–	T0.61	–
Proximal femur										
CTSA	Clarke (2023)	T17;R15	0.18	0.04	0.15	0.28	0.46	0.36	–	–
	Scheerlinck (2016)	T39;R39	0.05	0.04	0.03	0.04	0.08	0.06	–	–
Geomagic 7	Boettner (2015) ^a^	1) T30	–	–	–	–	–	–	–	0.23
		2) T30	–	–	–	–	–	–	–	0.18
		3) T30	–	–	–	–	–	–	–	0.20
	Boettner (2016)	T15	–	–	–	–	–	–	–	0.11
Tibia										
V3MA	De Laat (2024)	T9;R6	0.05	0.02	0.15	–	–	0.03	–	–
Glenoid										
CTMA	Brodén, Giles (2020)	T16;R12	0.23	0.17	0.20	0.44	0.48	0.71	–	–
micro-CT	Sukjamsri (2015)	T12	0.12	0.03	0.07	–	–	–	–	–
3D volume tool	Jun (2022)	T9;R3	0.06	0.24	0.15	–	–	0.11	–	–
Humerus										
CTMA	Brodén, Giles (2020)	T16;R12	0.11	0.07	0.09	0.34	0.32	0.22	–	–
Spine										
3D volume tool	Svedmark (2011)	T61;R61	0.57	0.28	0.71	1.00	0.57	0.28	–	–

For abbreviations, see Table 1.

n is the number of measurements with ‘T’ as translations and ‘R’ as rotations. Studies that used multiple protocols were named (1), (2), etc. Axis definition is reported according to the CT coordinate system: medial (X), posterior (Y), and proximal (Z). Measurements with unknown axis definition are depicted in U. Measurements in all axes are shown in mm for translations and degrees for rotations, and respectively in µm and millidegrees for micro-CT. If available, total translation (in mm) was inserted in TT.

a 3 different radiation protocols were used: (1) standard protocol for hip implants, (2) and (3) alternative low-dose protocols.

## Corrected version

**Table 2:** Reported accuracy of CT-RSA

Joint/Method	Author	n	Tx	Ty	Tz	Rx	Ry	Rz	U	TT
Acetabulum										
CTSA	Clarke (2023)	T17;R15	0.08	0.06	0.04	0.17	0.29	0.43	–	–
3D volume tool	Olivecrona (2003)	T30	–	–	–	–	–	–	T0.61	–
Proximal femur										
CTSA	Clarke (2023)	T17;R15	0.18	0.04	0.15	0.28	0.46	0.36	–	–
	Scheerlinck (2016)	T39;R39	0.05	0.04	0.03	0.04	0.08	0.06	–	–
Geomagic 7	Boettner (2015) ^a^	1) T30	–	–	–	–	–	–	–	0.23
		2) T30	–	–	–	–	–	–	–	0.18
		3) T30	–	–	–	–	–	–	–	0.20
	Boettner (2016)	T15	–	–	–	–	–	–	–	0.11
Tibia										
V3MA	De Laat (2024)	**T21;R21**	**0.19**	**0.04**	**0.13**	–	–	**0.04**	–	–
Glenoid										
CTMA	Brodén, Giles (2020)	T16;R12	0.23	0.17	0.20	0.44	0.48	0.71	–	–
micro-CT	Sukjamsri (2015)	T12	0.12	0.03	0.07	–	–	–	–	–
3D volume tool	Jun (2022)	T9;R3	0.06	0.24	0.15	–	–	0.11	–	–
Humerus										
CTMA	Brodén, Giles (2020)	T16;R12	0.11	0.07	0.09	0.34	0.32	0.22	–	–
Spine										
3D volume tool	Svedmark (2011)	T61;R61	0.57	0.28	0.71	1.00	0.57	0.28	–	–

For abbreviations, see Table 1.

n is the number of measurements with ‘T’ as translations and ‘R’ as rotations. Studies that used multiple protocols were named (1), (2), etc. Axis definition is reported according to the CT coordinate system: medial (X), posterior (Y), and proximal (Z). Measurements with unknown axis definition are depicted in U. Measurements in all axes are shown in mm for translations and degrees for rotations, and respectively in µm and millidegrees for micro-CT. If available, total translation (in mm) was inserted in TT.

a 3 different radiation protocols were used: (1) standard protocol for hip implants, (2) and (3) alternative low-dose protocols.

