# Supplementary material for: The accuracy and precision of CT-RSA in arthroplasty: a systematic review and meta-analysis
Source: Acta Orthop. 2025 Mar 28;96:295–303. doi: 10.2340/17453674.2025.43334 (PMC11971844; doi:10.2340/17453674.2025.43334)
Supplement: Supplementary file 1 [file ActaO-96-43334-s1.pdf]

Supplementary data

Figure 2. Forest plots of analyzed subgroups.

Figure 1a. The in-vitro precision of acetabulum (translation)

Study or Subgroup	Mean Difference	SE	Weight	Mean Difference IV, Random, 95% CI	Year
Olivecrona 2003	0.47	0.08	20.6%	0.47 [0.31, 0.63]	2003
Broden 2016	0.05	0.01	40.6%	0.05 [0.03, 0.07]	2016
Clarke 2023	0.09	0.02	38.8%	0.09 [0.05, 0.13]	2023
**Total (95% CI)**			**100.0%**	**0.15 [0.05, 0.25]**	

Heterogeneity: $Tau^2 = 0.01$; $Chi^2 = 29.40$, df = 2 ($P < 0.00001$); $I^2 = 93\%$
Test for overall effect: $Z = 2.96$ ($P = 0.003$)

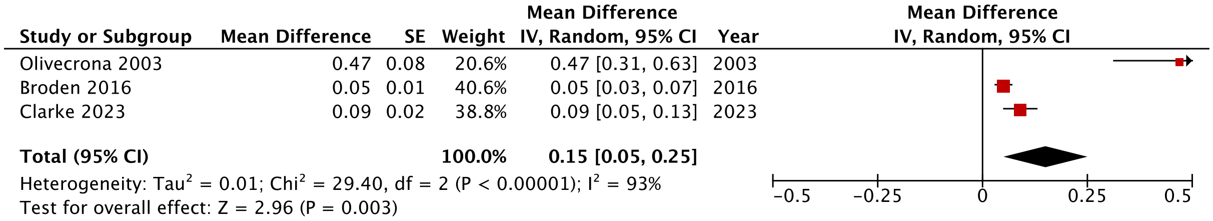

Figure 2b. The in-vitro precision of proximal femur (translation)

Study or Subgroup	Mean Difference	SE	Weight	Mean Difference IV, Random, 95% CI	Year
Scheerlinck 2016	0.01	0.004	34.9%	0.01 [0.00, 0.02]	2016
Eriksson 2019	0.18	0.03	33.2%	0.18 [0.12, 0.24]	2019
Clarke 2023	0.22	0.04	31.9%	0.22 [0.14, 0.30]	2023
**Total (95% CI)**			**100.0%**	**0.13 [−0.02, 0.28]**	

Heterogeneity: $Tau^2 = 0.02$; $Chi^2 = 58.08$, df = 2 ($P < 0.00001$); $I^2 = 97\%$
Test for overall effect: $Z = 1.76$ ($P = 0.08$)

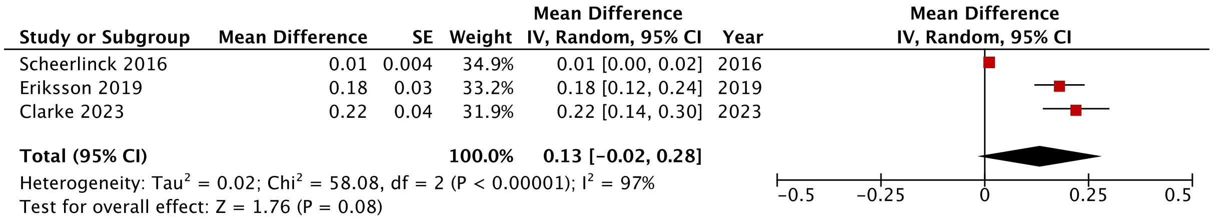

Figure 2c. The in-vitro precision of proximal femur (rotation)

Study or Subgroup	Mean Difference	SE	Weight	Mean Difference IV, Random, 95% CI	Year
Scheerlinck 2016	0.02	0.004	35.0%	0.02 [0.01, 0.03]	2016
Eriksson 2019	0.33	0.06	32.9%	0.33 [0.21, 0.45]	2019
Clarke 2023	0.39	0.07	32.1%	0.39 [0.25, 0.53]	2023
**Total (95% CI)**			**100.0%**	**0.24 [−0.03, 0.51]**	

Heterogeneity: $Tau^2 = 0.05$; $Chi^2 = 54.22$, df = 2 ($P < 0.00001$); $I^2 = 96\%$
Test for overall effect: $Z = 1.74$ ($P = 0.08$)

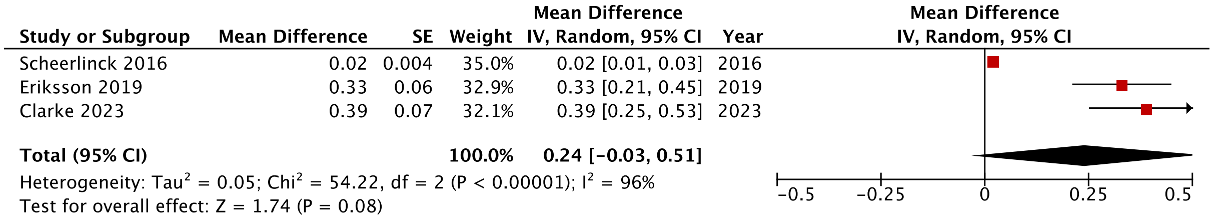

Figure 2d. The in-vitro precision of proximal tibia (translation)

Study or Subgroup	Mean Difference	SE	Weight	Mean Difference IV, Random, 95% CI	Year
Engseth 2023	0.07	0.005	33.4%	0.07 [0.06, 0.08]	2023
Øhrn 2023	0.01	0.001	34.0%	0.01 [0.01, 0.01]	2023
De Laat 2024	0.03	0.008	32.5%	0.03 [0.01, 0.05]	2024
**Total (95% CI)**			**100.0%**	**0.04 [−0.01, 0.08]**	

Heterogeneity: $Tau^2 = 0.00$; $Chi^2 = 143.28$, df = 2 ($P < 0.00001$); $I^2 = 99\%$
Test for overall effect: $Z = 1.72$ ($P = 0.09$)

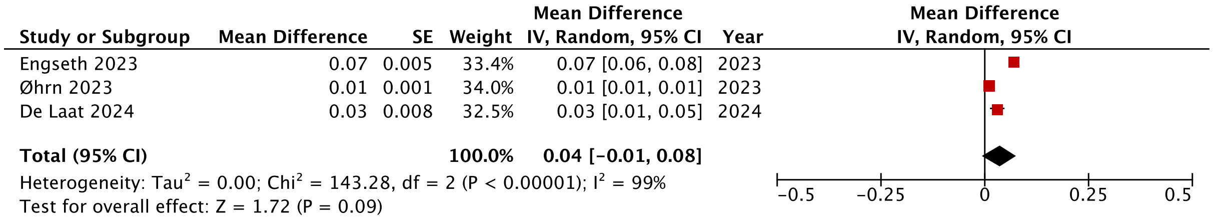

Figure 2e. The in-vitro precision of proximal tibia (rotation)

Study or Subgroup	Mean Difference	SE	Weight	Mean Difference IV, Random, 95% CI	Year
Engseth 2023	0.1	0.008	33.3%	0.10 [0.08, 0.12]	2023
Øhrn 2023	0.01	0.001	33.7%	0.01 [0.01, 0.01]	2023
De Laat 2024	0.11	0.01	33.0%	0.11 [0.09, 0.13]	2024
**Total (95% CI)**			**100.0%**	**0.07 [−0.00, 0.15]**	

Heterogeneity: $Tau^2 = 0.00$; $Chi^2 = 220.92$, df = 2 ($P < 0.00001$); $I^2 = 99\%$
Test for overall effect: $Z = 1.90$ ($P = 0.06$)

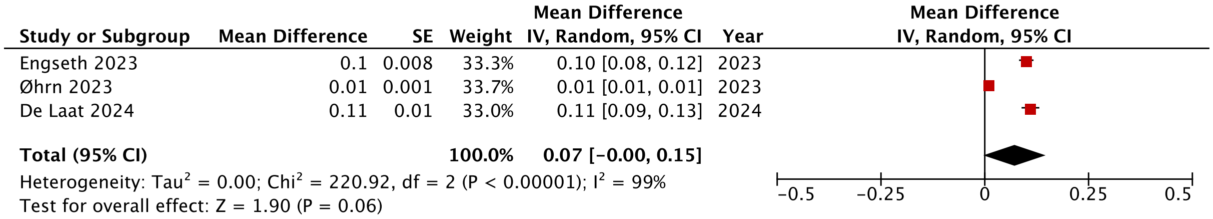

Figure 2f. The in-vivo precision of acetabulum (translation)

Study or Subgroup	Mean Difference	SE	Weight	Mean Difference IV, Random, 95% CI	Year	Mean Difference IV, Random, 95% CI
Broden, Sandberg 2020 (1)	0.17	0.03	10.4%	0.17 [0.11, 0.23]	2020	
Broden, Sandberg 2020 (2)	0.14	0.02	14.8%	0.14 [0.10, 0.18]	2020	
Broden, Sandberg 2020 (3)	0.19	0.03	10.4%	0.19 [0.13, 0.25]	2020	
Sandberg 2020	0.13	0.02	14.8%	0.13 [0.09, 0.17]	2020	
Stigbrand 2020	0.13	0.02	14.8%	0.13 [0.09, 0.17]	2020	
Broden 2021	0.13	0.02	14.8%	0.13 [0.09, 0.17]	2021	
Angelomenos 2022	0.09	0.01	19.9%	0.09 [0.07, 0.11]	2022	
**Total (95% CI)**			**100.0%**	**0.13 [0.11, 0.16]**		

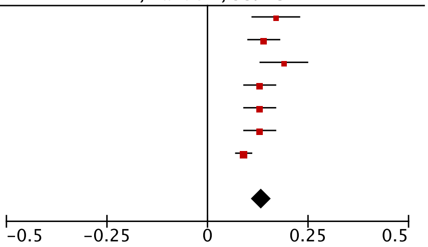

Heterogeneity: $Tau^2 = 0.00$; $Chi^2 = 18.89$, df = 6 (P = 0.004); $I^2 = 68\%$
Test for overall effect: Z = 10.12 (P < 0.00001)

Figure 2g. The in-vivo precision of acetabulum (rotation)

Study or Subgroup	Mean Difference	SE	Weight	Mean Difference IV, Random, 95% CI	Year	Mean Difference IV, Random, 95% CI
Broden, Sandberg 2020 (1)	0.33	0.05	13.4%	0.33 [0.23, 0.43]	2020	
Broden, Sandberg 2020 (2)	0.29	0.04	15.6%	0.29 [0.21, 0.37]	2020	
Broden, Sandberg 2020 (3)	0.24	0.03	17.8%	0.24 [0.18, 0.30]	2020	
Sandberg 2020	0.16	0.02	19.8%	0.16 [0.12, 0.20]	2020	
Broden 2021	0.26	0.04	15.6%	0.26 [0.18, 0.34]	2021	
Angelomenos 2022	0.3	0.03	17.8%	0.30 [0.24, 0.36]	2022	
**Total (95% CI)**			**100.0%**	**0.26 [0.20, 0.32]**		

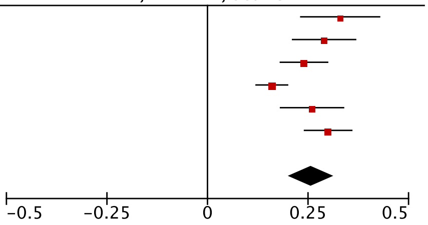

Heterogeneity: $Tau^2 = 0.00$; $Chi^2 = 24.60$, df = 5 (P = 0.0002); $I^2 = 80\%$
Test for overall effect: Z = 8.70 (P < 0.00001)

Table 7. Quality assessment using CASP

Author	Q1	Q2	Q3	Q4	Q5	Q6	Q7	Q8	Q9	Q10	Q11	Q12	Results
**Olivacrona (2003)**	1	1	1	1	N/A	1	0	1	0	1	1	1	82%
**Gortchacow (2011)**	1	1	1	0	N/A	1	0	0	1	0	1	0	55%
**Svedmark (2011)**	1	1	0	0	1	1	1	1	1	1	1	1	83%
**Gortchacow (2012)**	1	1	1	0	N/A	1	0	0	1	0	1	0	55%
**Boettner (2015)**	1	1	1	0	N/A	1	1	1	1	1	1	1	91%
**Sukjamsri (2015)**	1	1	1	0	N/A	1	0	0	1	0	1	0	55%
**(Scheerlinck (2016)**	1	1	1	0	0	1	1	1	1	1	1	1	83%
**Brodén (2016)**	1	0	1	0	N/A	1	1	0	1	1	1	1	73%
**Boettner (2016)**	1	1	1	0	N/A	1	1	1	1	1	1	1	91%
**Malfroy Camine (2016)**	1	1	1	0	N/A	1	1	1	1	0	1	0	73%
**Eriksson (2019)**	1	1	1	0	0	1	1	1	1	1	1	1	83%
**Brodén, Sandberg (2020)**	1	1	1	1	0	1	1	1	1	1	1	1	92%
**Brodén, Giles (2020)**	1	1	1	1	N/A	1	1	1	1	1	1	1	100%
**Sandberg (2020)**	0	1	1	1	1	1	1	1	1	1	1	1	92%
**Stigbrand (2020)**	1	1	1	1	1	1	0	1	1	1	0	1	83%
**Brodén (2021)**	1	1	1	1	1	1	1	1	1	1	1	1	100%
**Jun (2022)**	1	1	1	1	1	1	1	0	1	1	0	1	83%
**Angelomenos (2022)**	1	1	0	1	1	1	1	1	1	1	1	1	92%
**Clarke (2023)**	1	1	1	0	N/A	1	1	1	1	1	1	1	91%
**Engseth (2023)**	1	1	1	1	N/A	1	1	1	1	1	1	1	100%
**Øhrn (2023)**	1	1	1	1	N/A	1	1	1	1	1	1	1	100%
**Polus (2024)**	1	1	1	0	1	1	1	1	1	1	1	1	92%
**De Laat (2024)**	1	1	1	0	N/A	1	1	1	1	1	1	1	91%

Abbreviations: Q = Question, N/A = not applicable.

1 point was rewarded when the answer to the question was ‘yes’, or ‘no’ in case of questions regarding bias. 0 points were rewarded if the answer to the question was ‘no’, or ‘yes’ in case of questions regarding bias. Q2: an appropriate reference standard for accuracy was defined as a reliable indicator of 'true migration'. For precision tests, reliable double measurements with no migration, but with changing of position in between scans, was defined as an appropriate reference standard. Q5: spectrum bias included a clear description of the patient status. This question was only answered in in-vivo studies.

Search strings

Pubmed database 355 hits (21-10-2024):

(Arthroplasty[Mesh] OR arthroplast*[tiab] OR kneejoint*[tiab] OR hipjoint*[tiab] OR "joint prosthesis"[Mesh] OR "joint prosthesis"[title/abstract:~2] OR "joint prostheses"[title/abstract:~2] OR "prosthetic joint "[title/abstract:~2] OR "joints prosthesis"[title/abstract:~2] OR "joints prostheses"[title/abstract:~2] OR "prosthetic joints"[title/abstract:~2] OR "hip prosthesis"[Mesh] OR "hip prosthesis"[title/abstract:~2] OR "hip prostheses"[title/abstract:~2] OR "prosthetic hip "[title/abstract:~2] OR "hips prosthesis"[title/abstract:~2] OR "hips prostheses"[title/abstract:~2] OR "prosthetic hips"[title/abstract:~2] OR "knee prosthesis"[Mesh] OR "knee prosthesis"[title/abstract:~2] OR "knee prostheses"[title/abstract:~2] OR "prosthetic knee "[title/abstract:~2] OR "knees prosthesis"[title/abstract:~2] OR "knees prostheses"[title/abstract:~2] OR "prosthetic knees"[title/abstract:~2] OR "shoulder prosthesis"[Mesh] OR "shoulder prosthesis"[title/abstract:~2] OR "shoulder prostheses"[title/abstract:~2] OR "prosthetic shoulder "[title/abstract:~2] OR "shoulders prosthesis"[title/abstract:~2] OR "shoulders prostheses"[title/abstract:~2] OR "prosthetic shoulders "[title/abstract:~2] OR "joint implant"[title/abstract:~2] OR "joint implants"[title/abstract:~2] OR "joint replacement"[title/abstract:~2] OR "joint replacements"[title/abstract:~2] OR "joint reconstruction"[title/abstract:~2] OR "joint reconstructions"[title/abstract:~2]) AND ("Foreign-Body Migration"[Mesh] OR migrat*[Tiab] OR micromotion*[tiab] OR "motion analysis"[title/abstract:~2] OR "motion analyses"[title/abstract:~2] OR "motions analysis"[title/abstract:~2] OR "motions analyses"[title/abstract:~2] OR "CTRSA"[tiab] OR "CTMA"[tiab]) AND ("Tomography, X-Ray Computed"[Mesh] OR "computed tomography"[title/abstract:~2] OR "computed tomographies"[title/abstract:~2] OR "computed tomographic"[title/abstract:~2] OR "computerized tomography"[title/abstract:~2] OR "computerized tomographies"[title/abstract:~2] OR "computerized tomographic"[title/abstract:~2] OR CT[Tiab] OR CTbased[Tiab] OR CTscan*[Tiab] OR "CAT-Scan*"[tiab] OR "CATScan*"[tiab])

Embase database 371 hits (21-10-2024):

(exp "Arthroplasty"/ OR "arthroplast*".ti,ab. OR "kneejoint*".ti,ab. OR "hipjoint*".ti,ab. OR exp "joint prosthesis"/ OR (joint ADJ2 prosthesis) OR (joint ADJ2 prostheses) OR (prosthetic ADJ2 joint) OR (joint ADJ2 prosthesis) OR (joint ADJ2 prostheses) OR (prosthetic ADJ2 joint) OR (prosthetic ADJ2 joints) OR exp "hip prosthesis"/ OR (hip ADJ2 prosthesis) OR (hip ADJ2 prostheses) OR (prosthetic ADJ2 hip) OR (hips ADJ2 prosthesis) OR (hips ADJ2 prostheses) OR (prosthetic ADJ hips) OR exp "knee prosthesis"/ OR (knee ADJ2 prosthesis) OR (knee ADJ2 prostheses) OR (prosthetic ADJ2 knee) OR (knees ADJ2 prosthesis) OR (knees ADJ2 prostheses) OR (prosthetic ADJ2 knees) OR exp "shoulder prosthesis"/ OR (shoulder ADJ2 prosthesis) OR (shoulder ADJ2 prostheses) OR (prosthetic ADJ2 shoulder) OR (shoulders ADJ2 prosthesis) OR (shoulders ADJ2 prostheses) OR (prosthetic ADJ2 shoulders) OR (joint* ADJ2 implant*) OR (joint* ADJ2 replacement*) OR (joint* ADJ2 reconstruction*)) AND (exp "Foreign-Body Migration"/ OR migrat*.ti,ab. OR micromotion*.ti,ab. OR (motion ADJ2 analysis) OR (motion ADJ2 analyses) OR (motions ADJ2 analysis) OR (motions ADJ2 analyses) OR "CTRSA".ti,ab. OR "CTMA".ti,ab.) AND (exp "Tomography, X-Ray Computed"/ OR (computed ADJ2 tomograph*) OR (computerized ADJ2 tomograph*) OR CT.ti,ab. OR Ctbased.ti,ab. OR CTscan*.ti,ab. OR "CAT-Scan*".ti,ab. OR "CATScan*".ti,ab.) NOT ("Conference Abstract".pt. OR "Conference Paper".pt. OR "Conference Review".pt. OR congress*.ti. OR conference*.ti. OR "conference review*".ti,ab. OR "conference abstract*".ti,ab. OR "congress abstract*".ti,ab. OR abstract.ti. OR abstracts.ti.)

Cochrane database 40 hits (21-10-2024):

((arthroplast* OR kneejoint* OR hipjoint*):ti,ab,kw OR (joint NEXT/2 prosthesis) OR (joint NEXT/2 prostheses) OR (prosthetic NEXT/2 joint) OR (joint NEXT/2 prosthesis) OR (joint NEXT/2 prostheses) OR (prosthetic NEXT/2 joint) OR (prosthetic NEXT/2 joints) OR (hip NEXT/2 prosthesis) OR (hip NEXT/2 prostheses) OR (prosthetic NEXT/2 hip) OR (hips NEXT/2 prosthesis) OR (hips NEXT/2 prostheses) OR (prosthetic NEXT/2 hips) OR (knee NEXT/2 prosthesis) OR (knee NEXT/2 prostheses) OR (prosthetic NEXT/2 knee) OR (knees NEXT/2 prosthesis) OR (knees NEXT/2 prostheses) OR (prosthetic NEXT/2 knees) OR (shoulder NEXT/2 prosthesis) OR (shoulder NEXT/2 prostheses) OR (prosthetic NEXT/2 shoulder) OR (shoulders NEXT/2 prosthesis) OR (shoulders NEXT/2 prostheses) OR (prosthetic NEXT/2 shoulders) OR (joint* NEXT/2 implant*) OR (joint* NEXT/2 replacement*) OR (joint* NEXT/2 reconstruction*)) AND ((migrat* OR micromotion* OR "CTRSA" OR "CTMA"):ti,ab,kw OR (motion* ADJ2 analys*)) AND ((computed ADJ2 tomograph*) OR (computerized ADJ2 tomograph*) OR (CT OR Ctbased OR CTscan* OR CAT-Scan* OR CATScan*):ti,ab,kw)
